# Serum Procalcitonin is a sensitive and specific marker in the diagnosis of septic arthritis and acute osteomyelitis

**DOI:** 10.1186/1749-799X-8-19

**Published:** 2013-07-04

**Authors:** Karthikeyan Maharajan, Dilip Kumar Patro, Jagdish Menon, Ananthanarayanan Palghat Hariharan, Sabhash Chandra Parija, Murali Poduval, Sreenivas Thimmaiah

**Affiliations:** 1 Department of Orthopedics, JIPMER, Puducherry, India; 2 Department of Biochemistry, JIPMER, Puducherry, India; 3 Department of Microbiology, JIPMER, Puducherry, India

**Keywords:** Serum, Procalcitonin, Septic, Arthritis, Acute, Osteomyelitis, Diagnosis, Inflammatory, Marker

## Abstract

**Background:**

Early diagnosis of Acute Osteomyelitis (OM) and Septic Arthritis (SA) is of vital importance to avoid devastating complications. There is no single laboratory marker which is sensitive and specific in diagnosing these infections accurately. Total Count, ESR and CRP are not specific as they can also be elevated in non pyogenic causes of inflammation. Pus Culture and sensitivity is not a true gold standard due to its varied positivity rates (40 – 70%). Serum Procalcitonin (PCT), at *0.5 ng/ml* is found to be an accurate marker for pyogenic infections. The objectives of this study were to show that PCT is an accurate marker in differentiating Acute Osteomyelitis and Septic Arthritis from viral and non infective inflammatory bone and joint conditions.

**Methods:**

Patients of all age groups (n = 82) with suspected Acute Osteomyelitis and Septic Arthritis were prospectively included in this study. All patients were subjected to TC, CRP, PCT, IgM Dengue, IgM Chikungunya, pus and blood culture and sensitivity. At the end of the study, patients were classified into 3 groups: Group 1 = Confirmed Pyogenic (n = 27); Group 2 = Presumed Pyogenic (n = 21); Group 3 = Non – infective inflammatory (n = 34).

**Results:**

Group 1 has higher mean PCT levels than Group 2 and 3 (*p < 0.05*). PCT, at *0.4 ng/ml*, was 85.2% sensitive and 87.3% specific in diagnosing Septic Arthritis and Acute Osteomyelitis. In comparison, PCT at conventional cut – off of *0.5 ng/ml* is 66.7% sensitive and 91% specific.

**Conclusion:**

Serum Procalcitonin, at a cut – off of *0.4 ng/ml*, is a sensitive and specific marker in the diagnosis of Septic Arthritis and Acute Osteomyelitis.

## Background

Acute bone and joint infections are truly a diagnostic enigma in the Emergency Department. Management of these focuses on providing a rapid etiologic diagnosis since therapies and outcome vary widely depending upon the cause. Delayed diagnosis and treatment leading to disabling sequelae are not uncommon. Functional disabilities occur in 25 to 50% of cases and can even be life threatening in 5 to 15% of cases [1-3]. Total Count (TC), Erythrocyte Sedimentation Rate (ESR) and C – Reactive Protein (CRP) are routinely used in the diagnosis of these infections but no specific laboratory test exists with the exception of isolation of pathogenic organism from the bone or synovial fluid [4,5]. A positive culture result has high specificity but even this cannot be considered as gold standard because it lacks sensitivity (only 40-60%) and the results are available only after 2 to 3 days [2,3]. Despite this fact, culture is being used by many researchers as positive gold standard and patients without any clinical evidence plus negative culture as negative gold standard. The lack of sensitive laboratory markers or a gold standard investigation for diagnosing bacterial infections clinically has contributed to the overuse of antibiotics especially in neonates and elderly patients where clinical signs could be very subtle. The concept of providing antibiotics for every suspected infection is slowly being withdrawn because of emerging issues with antimicrobial resistance.

In this regard, there is a need for a biochemical marker which shows high sensitivity and specificity in diagnosing infection and also as a guide for starting antibiotics. There is now enough evidence to support the role of Procalcitonin (PCT) as a diagnostic and prognostic marker in infective conditions with its very high specificity for bacterial infections [6-9]. On the contrary, there are only very few studies evaluating its efficacy in Septic Arthritis (SA) and Acute Osteomyelitis (OM) with varying results [10-14]. Serum levels of Procalcitonin is very low in healthy individuals (< 0.1 ng/ml) and increases rapidly in response to bacterial endotoxin [15,16]. These properties together with a half life of 22 to 29 hours have made Procalcitonin, a convenient tool to monitor serious infections and to discriminate bacterial infections from viral and non infective inflammatory conditions [17,18]. Owing to its high specificity, Procalcitonin can also be used as a guide for starting antibiotics and monitoring treatment [19,20].

Hence this study was conducted prospectively with the *objectives to show that PCT is an accurate marker in the diagnosis of Acute Osteomyelitis and Septic Arthritis. Early treatment can be started which can decrease the incidence of disabling sequelae.*

## Materials and methods

This study was conducted in the Department of Orthopaedics, in a 1200 bedded tertiary health – care centre from January 2010 to June 2011. We included all cases of suspected acute (less than 2 weeks) synovitis / arthritis and osteomyelitis of all age groups with no evidence of infection elsewhere. We excluded (1) all cases of chronic arthritis and chronic osteomyelitis; (2) cases where antibiotics were given before presenting to us. (3) cases with foci of infection elsewhere. (4) immunocompromised hosts. The study was approved by Institute Research council and Ethics Committee and conducted in accordance to the standards of Declaration of Helsinki, 1964. Detailed informed consent was obtained from all patients or from parents and legal guardians included in this study.

All patients were assessed for clinical parameters which included presence of swelling; joint effusion; tenderness; local warmth; deformity; range of movement restriction and presence of septicemic symptoms. Detailed proforma was used to record patient details and clinical findings. Plain radiography [Anteroposterior and Lateral views] and Ultrasound (USG) of the involved bone and joint were done. Under adequate anesthesia and proper aseptic precautions, Aspiration was done using 18G needle from the joint and bone with subperiosteal abscess. In the absence of subperiosteal abscess, aspirate was obtained by drilling the bone with 2 mm drill bit under anesthesia. This was followed by definitive surgery in the form of arthrotomy or incision and drainage (I&D).

Aspirate was immediately processed for the presence of pus cells, gram staining, culture and sensitivity (C/S). All patients were subjected to Blood C/S. Culture and sensitivity was considered as the gold standard in this study. Laboratory analyses included estimation of ESR, TC, CRP, Procalcitonin, IgM Dengue and IgM Chikungunya. ESR was estimated using Wintrobe’s method. CRP was measured using Nephelometer [BNPROSPEC, Germany]. The cut-off in our laboratory was 3.02 mg/l above which CRP was considered positive. PCT was determined by Quantitative Immunoluminetric test [LUMItest, BRAHMS Diagnostica, Berlin, Germany) by the investigator who followed the assay manufacturer’s recommendations. The threshold for PCT detection defined by our laboratory is 0.1 *ng*/ml. Samples were analyzed for IgM Dengue and IgM Chikungunya using ELISA technique. Dengue and Chikungunya are the commonest viral infections presenting with bone and joint symptoms in our region and the only feasible tests available in our institution. They were performed to rule out viral etiology for acute bone and joint infective conditions.

At the end of the study, patients were divided into three groups: Confirmed Pyogenic group (Group 1), Presumed Pyogenic group (Group 2) and Non-Infective inflammatory group (Group 3) based on the above parameters. In Group 1, there was a high clinical suspicion of infection and pus was aspirated. Pus and/or blood culture was positive for bacteria and patients received full course of antibiotics [2 weeks of sensitive IV antibiotics and 6 weeks of oral antibiotics]. This group included Culture Positive Septic Arthritis (Group 1a) and Culture Positive Acute Osteomyelitis (Group 1b). In Group 2, there was clinical suspicion of infection and altered synovial fluid was aspirated while aspirate / blood culture remained negative and these patients too were treated with full course of antibiotics due to clinical suspicion of infection. This group included culture negative septic arthritis (Group 2a) and culture negative acute osteomyelitis (Group 2b). In Group 3, patients had acute symptoms pertaining to bone and joint but there was no suspicion of infection clinically (acute synovitis due to rheumatoid arthritis, juvenile rheumatoid arthritis, gout, trauma, transient synovitis of hip, sickle cell bone crisis and non-specific causes). Synovial fluid was aspirated and aspirate culture remained negative and hence these patients were not started on any antibiotics (Table 1).

**Table 1:** Patient Characteristics n = 82

**Parameter**	**Value**
Symptom duration (days)^*^	3 (2–5)
Males / Females	58 / 24
Age (years) ^¥^	25.33 (10 days – 86 years)
Group	
Confirmed pyogenic (C+SA / C+OM)	27 (19 / 8)
Presumed pyogenic (C-SA / C-OM)	21 (17 / 4)
Non – infective inflammatory	34
RA	8
Juvenile RA	4
Gout	2
Transient synovitis of hip	5
Trauma	7
Sickle cell crisis	1
Non – specific synovitis^ф^	7
Blood C / S	3 (11.11%)
Pus C / S (C+SA / C+OM)	27 (19 / 8)
Methicillin Resistant SA	20 (12 / 8)
Staphylococcus aureus	3 (3 / 0)
Coagulase negative SA	1 (1 / 0)
Pseudomonas aeruginosa	1 (1 / 0)
Klebsiella pneumonia	1 (1 / 0)
Streptoccus species	1 (1 / 0)

* values expressed as median; ¥ value expressed as mean; ф Non specific synovitis = where cause cannot be found; *RA* Rheumatoid Arthritis, *C+SA* culture positive Septic arthritis, *C+OM* culture positive acute osteomyelitis, *C-SA* culture negative Septic Arthritis, *C-OM* culture negative acute osteomyelitis, *C/S* culture & sensitivity, *SA* Staphylococcus aureus.

Mean levels of TC, ESR, CRP and PCT were compared between these groups and sensitivity, specificity and predictive values of PCT were assessed.

The statistical significance of all parameters (TC, ESR, CRP and PCT) were analyzed using *Independent Students T test* and *One Way ANOVA* with *BonFerroni PostHoc test*. The sensitivity, specificity and predictive values were analyzed using *SPSS software version 19. Newcombe* method was used to calculate 95% confidence intervals (CI). *p <* 0.05 was considered statistically significant.

## Results

A total of 106 patients presented with clinical presentations suggestive of OM and SA. Out of these, 24 patients were excluded from the study as 11 patients gave a history of antibiotic administration before presentation and 13 patients had other foci of infection. The study group included 82 patients of all age groups (Table 1). The youngest was a 10 days old neonate and the oldest was 86 years of age with the mean age of 25.33 years. 23 patients (28.04%) were less than 5 years of age and 28 patients (34.14%) were above 40 years. Out of 82 patients, there were 58 males (70.73%) and 24 females (29.26). Blood culture was positive (11.11%) for MRSA in three cases, two cases of SA involving Hip joint and one case of OM of Distal Femur. MRSA was the commonest organism isolated from pus. It was positive in 74.07% of patients in Group 1; 63.16% in Group 1a and 100% in Group 1b. Viral analysis for IgM Dengue and Chikungunya did not yield any positive results.

All data in this study follow Normal type of distribution. On comparison of mean (Table 2), Group 1 had higher mean PCT values (1.0005) than group 2 and 3 which is statistically significant (*p = 0.001*). On multivariate analysis, Group 1b has higher mean PCT values (0.8453) than Group 1a, but not statistically significant (*p = 0.76*). Group 2a has higher mean PCT levels (0.5594) than Group 2b with no statistical significance (*p = 0.32*). Patients with positive blood culture had higher mean PCT levels (1.68) but not statistically significant (*p = 0.138*). Patients who grew MRSA in the aspirate had statistically significant (*p = 0.01*) higher mean PCT levels (1.02). Similarly, patients who had positive X-ray changes [lytic lesions, erosions, dislocation] and positive ultrasound findings [presence of moving echoes in joint / subperiosteal abscess] had statistically significant higher mean PCT levels 0.82 (*p = 0.001*) and 1.06 (*p = 0.001*) respectively.

**Table 2:** Mean with SD for all parameters

**Parameter**	**Group**	**N**	**Mean**	**S.D**	**P – value**
**TC**	Confirmed	27	9311.11	1531.05	**0**
	Presumed	21	8600	1063.01	
	Non-pyogenic	34	7279.41	1328.65	
**ESR**	Confirmed	27	61.85	10.55	**0**
	Presumed	21	51.57	12.96	
	Non-pyogenic	34	21.53	11.778	
**CRP**	Confirmed	27	12.35	5.77	**0.014**
	Presumed	20	11.31	4.56	
	Non-pyogenic	9	6.44	3.74	
**PCT**	Confirmed	27	1.00	0.73	**0**
	Presumed	21	0.53	0.46	
	Non-pyogenic	34	0.15	0.05	

*TC* Total Count, *ESR* Erythrocyte Sedimentation Rate, *CRP* C – Reactive Protein,

*PCT* Procalcitonin.

On analysis of other parameters, the mean TC and ESR were found to have raised in Group 1 and 2 with statistical significance (*p < 0.05*). In contrast, Group 1 had higher CRP levels than Group 2 which is not statistically significant (*p = 0.1*).

Sensitivity and specificity PCT at various cut – offs is depicted in Table 3. For Confirmed versus Presumed / Non – infective groups, at a cut off of 0.5 ng/ml, PCT is found to be 66.7% sensitive and 91% specific (PPV = 78.2%; NPV = 84.7%) in diagnosing culture positive pyogenic infections. But at 0.4 ng/ml, it is 85.2% sensitive and 87.3% specific (PPV = 76.6%; NPV = 92.3%). Levels lower than this has high sensitivity but low specificity and vice versa. So a cut off at which sensitivity and specificity got balanced was taken in this study. For Confirmed / Presumed versus Non – infective groups, PCT is 47.9% sensitive and 100% specific (PPV = 100%; NPV = 57.6%) in diagnosing clinically suspected infection at 0.5 ng/ml while it is 62.5% sensitive and 100% specific (PPV = 100%; NPV = 65.4%) at 0.4 ng/ml. For confirmed pyogenic versus non-pyogenic, at a cut – off of 0.4 ng/ml, PCT is 85.17% sensitive and 100% specific with PPV and NPV of 100% and 93.8% respectively (Table 4).

**Table 3:** Sensitivity and Specificity of PCT at various cut - offs

**PCT level**	**Confirmed / presumed Vs non infective**		**Confirmed Vs presumed / infective**	
	**Sensitivity %**	**Specificity %**	**Sensitivity %**	**Specificity %**
0.1	100	9.1	100	14.7
0.21	96.3	56.4	97.9	91.2
0.27	88.9	67.3	85.4	97.9
0.3	88.9	74.5	77.1	97.9
0.32	88.9	81.8	68.8	97.9
0.34	83.7	83.6	66.7	100
**0.4**	**85.2**	**87.3**	**62.5**	**100**
0.45	74.4	90.9	50	100
**0.5**	**66.7**	**90.9**	**47.9**	**100**
0.6	55.6	90.9	41.7	100
0.7	51.9	90.9	37.5	100
0.8	51.9	92.7	35.4	100
0.9	48.1	94.5	33.3	100
1	44.4	94.5	31.3	100

**Table 4:** Sensitivity, Specificity and Predictive values of PCT at 0.5 and 0.4 ng/ml

**Groups**	**PCT (ng/ml)**	**Sensitivity % 95% CI**	**Specificity % 95% CI**	**PPV % 95% CI**	**NPV % 95% CI**	**LR**
Confirmed Pyogenic vs presumed + Non – pyogenic	0.5	66.7 (46 – 83.4)	91 (80 – 97)	78.2 (56 – 92.5	84.7 (73–92.5)	7.33
	0.4	85.2 (66.3 – 95.8)	87.3 (75.5 – 94.7)	76.6 (57.7 –90.1)	92.3 (81.5 - 98)	6.69
Confirmed+ Presumed vs non – pyogenic	0.5	47.9 (33.2 – 62.8)	100 (89.7 – 100)	100 (85.2 -100)	57.6 (44.1 -70.4)	*
	0.4	62.5 (47.4 – 76)	100 (89.7 – 100)	100 (88.4 – 100)	65.4 (60 – 78)	*
Confirmed vs non – pyogenic	0.5	66.67 (46 – 83.4)	100 (91.2 - 100)	100 (87.4 – 100)	86.3 (77.8 – 94.9)	*
	0.4	85.19 (66.3 – 95.8)	100 (90.2 – 100)	100 (88.7 – 100)	93.8 (82.7 - 96.5)	*

*PCT* Procalcitonin, *PPV* Positive Predictive Value, *NPV* Negative Predictive Value, *CI* Confidence Interval, *LR* Likelihood Ratio, * LR cannot be calculated because of zero value.

**Figure 1:**
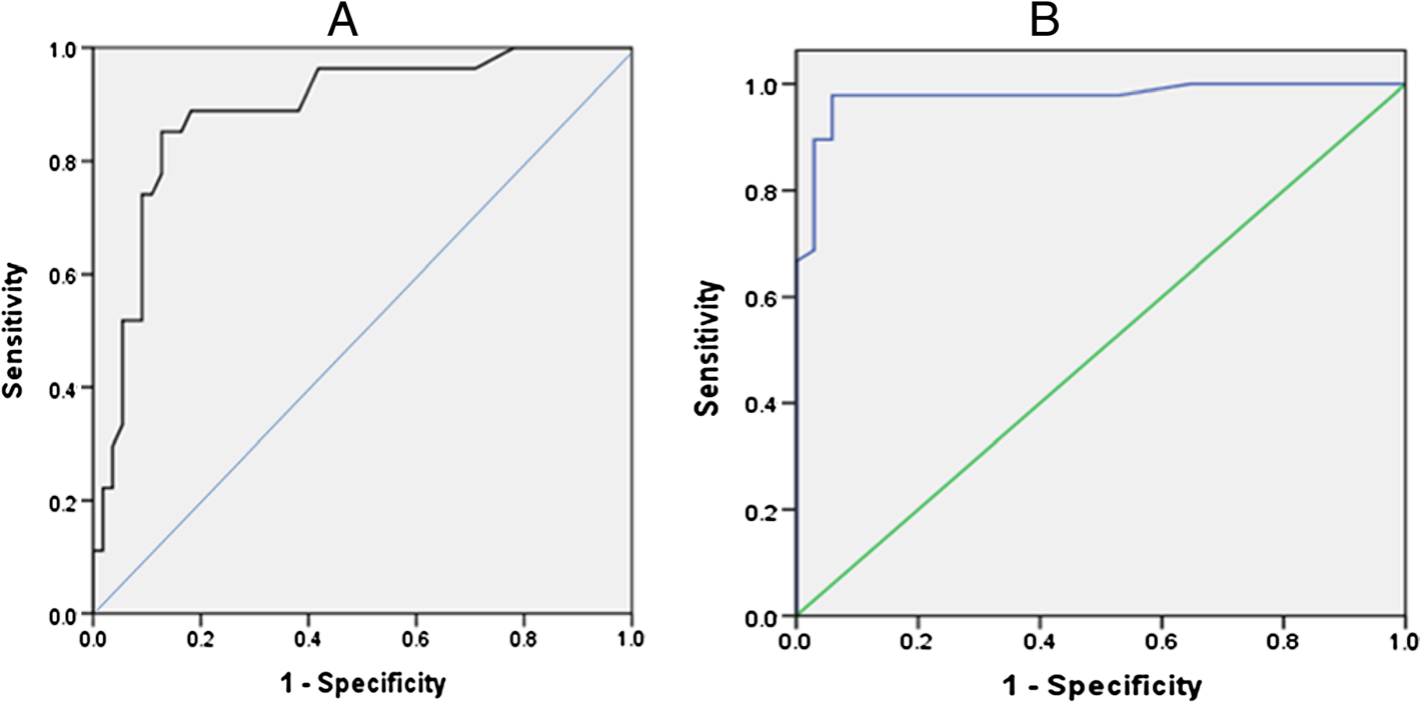
ROC for PCT.

Area Under the Curve (AUC) on plotting Receiver Operating Curves (ROC) was 0.886 (S.E= 0.04) for Confirmed versus Presumed / Non – infective groups while AUC is 0.976 (S.E = 0.016) for Confirmed / Presumed versus Non – infective groups (Figure 1).

## Discussion

Septic Arthritis and Acute Osteomyelitis are relatively common entities in day to day orthopedic practice and in a tertiary referral centre like ours, it is more common. But the diagnosis of these infections faces the following problems: inadvertent use of antibiotics by the physician who first sees the patient before the proper diagnosis is made; pus culture and sensitivity which is often considered the gold standard is not a useful gold standard because of its low positivity rates; absence of a single laboratory parameter with high specificity and sensitivity; dubious presentations of these infections in the very young and the old; hence the search for a realistic laboratory marker is essential.

Many of the complications secondary to delayed diagnosis have come down in number due to the availability of powerful antibiotics but on the other hand this has led to the emergence of anti- microbial resistance due to its inadvertent and irrational use. This is of serious concern because practically only few drugs are available as of now to tackle serious infections [21-23]. PCT has been found to be a promising marker in diagnosing bacterial infections with its high specificity. Its usefulness over markers like TC, ESR and CRP has been described in several conditions like sepsis, upper respiratory tract infections, pneumonias, pancreatitis, pyelonephritis, burns and in various other conditions [18,24,25]. With this background, this study was conducted with the following objectives: To show that PCT is an accurate marker for differentiating OM and SA from viral infections and non infective inflammatory bone and joint conditions and to evaluate Sensitivity, Specificity and Predictive values of Serum Procalcitonin in diagnosing OM and SA.

Pus culture is expected to be positive in 40 to 60% in patients of SA and OM though it is 100% specific [2,3]. In the present study, pus culture positivity is 56.25%. Staphylococcus aureus was described as the most common identifiable causative organism which accounts for more than 50% of isolated organism in acute hematogenous osteomyelitis and 30% in septic arthritis. MRSA is the commonest organism in the present study isolated in 74.07% of cases (63.16% of septic arthritis cases and 100% of acute osteomyelitis). Serum PCT level less than 0.5 ng/ml is considered normal [15,17]. However, there is no unanimous agreement in deciding the cut – off because PCT is an emerging diagnostic marker and is either undetectable or very low in healthy individuals. Studies by Butbul Aviel et al., [10]; Fottner et al., [11]; Martinot et al., [13] and Faesh et al., [14] have taken 0.5 ng/ml as cut – off above which it is considered as a marker of pyogenic infection. However, study by Hogle et al., [12] has taken 0.25 ng/ml as the cut-off. This reflects the absence of a general consensus in deciding the cut-off.

**Figure 2:**
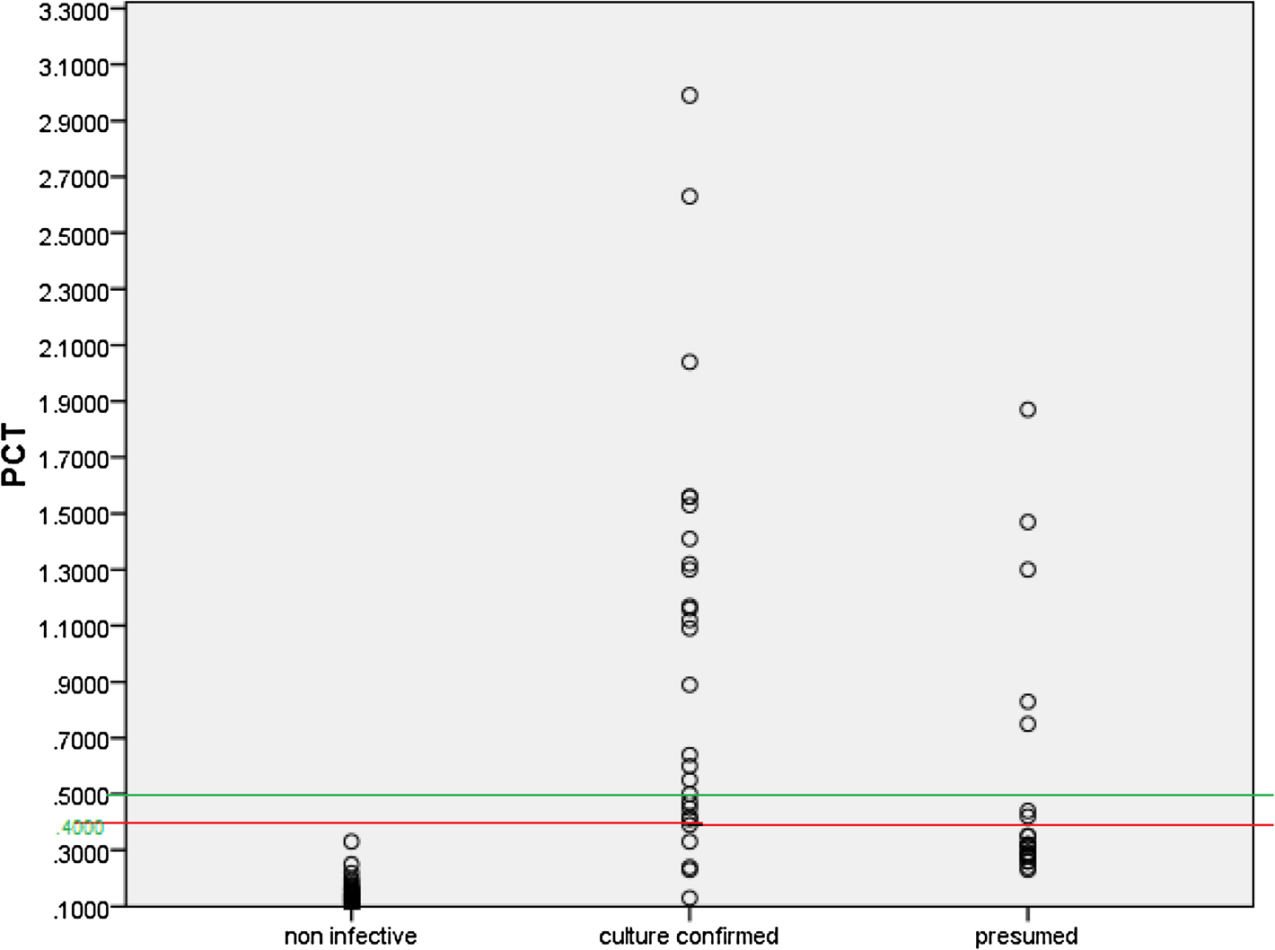
Scatter diagram plotting the distribution of patients with respect to PCT cut-offs.

In the present study, the sensitivity, specificity, positive predictive value and negative predictive value of PCT was assessed at 0.5 ng/ml and 0.4 ng/ml. For confirmed pyogenic versus presumed / non pyogenic, at 0.4 ng/ml, PCT is 85.2% sensitive and 87.3% specific in diagnosing culture positive SA and OM (Figure 2) with PPV and NPV of 76.6% and 92.3% respectively. The balancing of sensitivity and specificity is essential for testing any new diagnostic marker and hence 0.4 ng/ml is taken as the cut-off in this study. For confirmed / presumed pyogenic versus non-pyogenic, at a cut – off of 0.4 ng/ml, PCT is 62.5% sensitive and 100% specific in diagnosing clinically positive infection with PPV and NPV of 100% and 65.4% respectively. For confirmed pyogenic versus non-pyogenic, at a cut – off of 0.4 ng/ml, PCT is 85.17% sensitive and 100% specific in diagnosing clinically positive infection with PPV and NPV of 100% and 93.8% respectively.

There are only few studies evaluating the efficacy of PCT in SA and OM. Butbul Aviel et al., [10] in 2005, have shown that PCT at 0.5 ng/ml was a poorly sensitive but highly specific marker using a semi quantitative PCT card test with higher sensitivity for OM than SA. However, we have not attempted to analyze separately for SA and OM as PCT as a marker can diagnose only the presence and severity of infection and is not influenced by the site of infection. Similarly, studies by Fottner et al., [11]; Martinot et al., [13] and Sabine Faesch et al., [14] have shown PCT as a poorly sensitive marker with high specificity at a cut-off of 0.5 ng/ml. This could be due to the low sample size as reflected in all these studies. The study by Hogle et al., [12] has shown PCT as a highly sensitive marker but low specific marker at a cut-off of 0.25 ng/ml. The present study has included 36 patients of SA and 12 patients of OM which is considerably higher when compared to other studies. The present study has shown equally high sensitivity and specificity when compared to other groups. The strengths of the present study are: Prospective study; the number of patients with septic arthritis and acute osteomyelitis are more when compared to other studies; this study has included patients of all age groups; the LUMItest [BRAHMS Diagnostica] used has a very low detection limit of 0.1 ng/ml. The limitation of our study is that overall sample size is low but not to extent of affecting statistical analysis. We have included patients all age groups (10 days old to 86 years). Though this might have an impact on sensitivity and specificity rates, majority of patients (66%) in our study were less than 40 years. Through this study, we would also like to show that adult septic arthritis is not an uncommon entity, atleast in our parts of the world. Ours is a tertiary referral hospital catering to different referral patterns. Though this might have an impact on the result of this study, it is less likely as we stringently followed our inclusion criteria. We have included only cases of dengue and chikungunya as these are the only relevant viral infections common in our region but these may not be common in other parts of the world. The present study was designed to assess the diagnostic value of PCT. Hence serial PCT measurements which will help us to study its prognostic significance were not done.

## Conclusion

The present study has shown that Serum Procalcitonin, at a cut – off of 0.4 ng/ml, is a sensitive and specific marker in the diagnosis of Acute Osteomyelitis and Septic Arthritis. This is in comparison to the conventional cut – off of 0.5 ng/ml which is specific but less sensitive. Thus, Serum Procalcitonin may be used as a new diagnostic marker for initiation of treatment in the management of Acute Osteomyelitis and Septic arthritis.

## Abbreviations

SA: Septic arthritis; OM: Acute osteomyelitis; TC: Total count; ESR: Erythrocyte sedimentation rate; CRP: C – reactive protein; PCT: Procalcitonin.

## Competing interests

The authors declare that they have no competing interests.

## Authors’ contributions

KM conducted the study. DKP, JM and MP guided in grouping the patients, AHP in Procalcitonin estimation, SCP in viral markers analysis, STM in the preparation of this manuscript. All authors have read and approved the final version of this manuscript.
